# Orthogonal Functionalization of Oxo‐Graphene Nanoribbons

**DOI:** 10.1002/chem.202403645

**Published:** 2024-12-02

**Authors:** Lucia Merkel, Christof Neumann, Christian E. Halbig, Anton Habel, Xin Chen, Andrey Turchanin, Siegfried Eigler

**Affiliations:** ^1^ Institute of Chemistry and Biochemistry Freie Universität Berlin Altensteinstraße 23a 14195 Berlin Germany; ^2^ Institute of Physical Chemistry Friedrich Schiller University Jena Lessingstraße 10 07743 Jena Germany

**Keywords:** Graphene, Nanoribbons, Nanostructures, Orthogonal functionalization

## Abstract

Oxo‐graphene nanoribbons (oxo‐GNRs) can be prepared by the oxidative unzipping of single‐walled carbon nanotubes. We present an orthogonal functionalization method for the functionalization of the rims and the π‐surface, respectively, what is only possible due to the high rim portion in oxo‐GNRs. In particular, X‐ray photoelectron spectroscopy could be used to follow the reaction and detect the marker atoms contained in the addends. We propose that the reported orthogonal functionalization strategy can also be applied on other oxo‐functionalized carbon materials, such as graphene quantum dots, or reduced graphene oxide flakes.

## Graphical abstract

Oxo‐graphene nanoribbons (oxo‐GNRs), created by unzipping single‐wall carbon nanotubes (CNTs), are orthogonally functionalized at their rims and π‐surfaces using hydrazine derivatives and diazonium salts. This study highlights successful sequential functionalization of up to 40 % edge atoms and 4 % basal plane atoms. Such modifications enhance oxo‐GNRs’ potential in nanoelectronics and offer broader application possibilities for oxo‐functionalized materials.

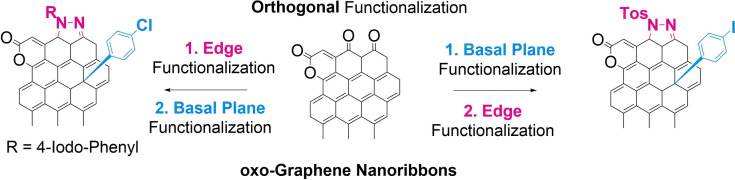

## Introduction

**Figure 1:**
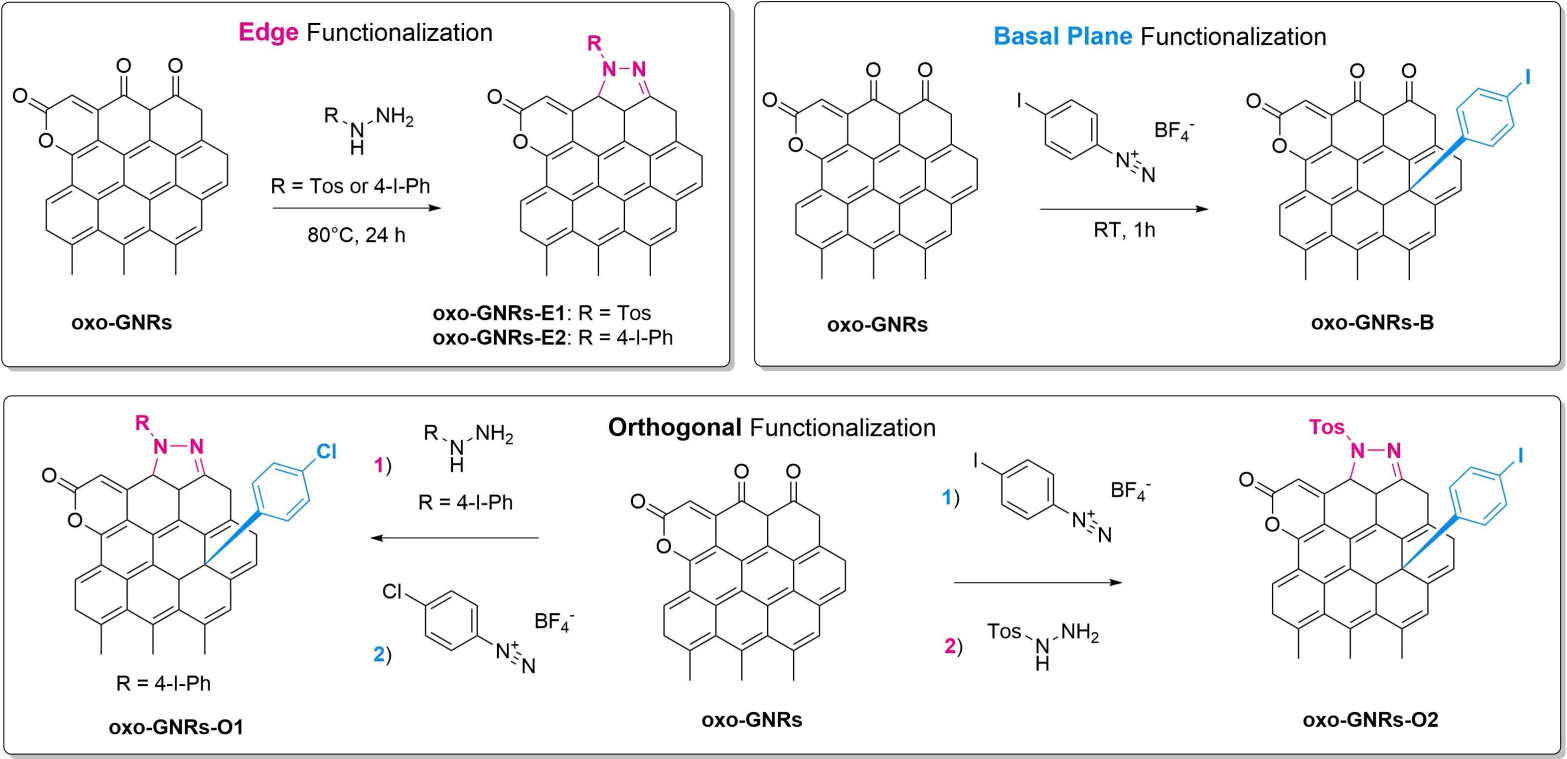
Functionalization of oxo‐GNRs with hydrazine derivatives on the edges (top left), diazonium salts on the basal plane (top right) and orthogonal (bottom). The reaction conditions for the individual steps of the orthogonal synthesis remain the same as shown in the top panels for the edge and basal plane functionalization and are omitted for clarity in the bottom panel.

Graphene nanoribbons (GNRs), quasi one‐dimensional (1D) materials, are gaining attention for their unique electronic properties including a bandgap, which is absent in graphene. This bandgap is not solely determined by the width of the ribbons^[1]^ but also heavily influenced by their geometrical edge structure[^2^, ^3^] and attached functional groups.[^4^, ^5^] Hence, edge engineering^[6]^ is a promising approach to tailor the GNRs’ electronic properties to meet the requirements for specific applications such as nanoelectronic devices^[7]^ or logic circuits.^[8]^ While a bottom‐up approach allows for extensive control over the edge structure to achieve atomically precise zigzag or armchair edges, as demonstrated by Müllen *et al*.,[^9^, ^10^] such control over the edge structure is hardly achievable by the oxidative unzipping of carbon nanotubes (CNTs). Unzipping allows, however, large‐scale production of oxo‐GNRs with various oxo‐groups at the ribbon edges, such as 1,3‐diketones and lactones, which serve as a starting point for post‐functionalization. Tour *et al*. reported that the modified Hummers method for oxidative unzipping of CNTs yields these oxo‐groups.^[11]^ This allows for functionalization of these moieties with hydrazine, as shown by Park *et al*. for oxo‐graphene.^[12]^ We have previously reported on the oxidative unzipping of single‐wall CNTs, which results in an intact sp^2^‐lattice of the resulting oxo‐GNRs and oxo‐groups at rims.^[13]^ The π‐surface can be functionalized with diazonium salts, derived from the knowledge of graphene functionalisation.[^14^, ^15^, ^16^] Here, we demonstrate the orthogonal functionalization of oxo‐GNRs produced by unzipping of single‐wall CNTs. Hydrazine derivatives are reacted with oxo‐groups at rims of oxo‐GNRs and diazonium salts react with the π‐surface as shown in Figure 1. We propose that substituted pyrazoline and aryl moieties on the ribbons’ edges and basal plane are formed, respectively. We selected hydrazine derivatives and diazonium salts containing different heteroatoms, which serve as probes in X‐ray photoelectron spectroscopy (XPS) measurements, indicating successful functionalization.

## Results and Discussion

**Figure 2:**
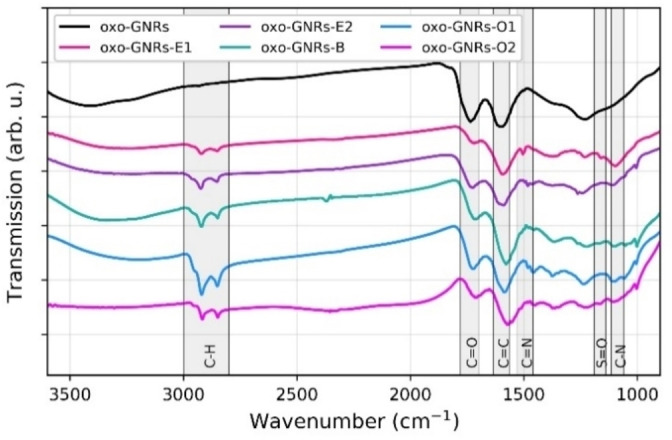
FTIR spectra of oxo‐GNRs and the corresponding functionalization products.

Oxo‐graphene nanoribbons were synthesized by oxidatively unzipping (6,5)‐single‐walled carbon nanotubes using potassium permanganate in a sulfuric acid medium, as reported before.^[13]^ This method enabled the formation of narrow, oxidized nanoribbons with widths ranging from 2 nm to 4 nm, while effectively preserving the honeycomb lattice structure in the central regions of the ribbons. Photoluminescence measurements verified the preservation of an intact π‐system in the ribbon cores. Oxidation was primarily localized at the ribbon edges, resulting in functional groups such as lactones and 1,3‐diketones at the rims, with a typical C/O ratio of approximately 3. Edge functionalization was achieved by adding a phenylhydrazine derivative (4‐iodophenylhydrazine or tosylhydrazine) to an aqueous oxo‐GNR dispersion and stirring at 80 °C for one day. For basal plane functionalization, an ice‐cooled oxo‐GNR dispersion was mixed with acetone to solubilize the 4‐iodobenzediazonium salt and then stirred for 1 hour. All samples were then purified by ultracentrifugation and dialysis. For the orthogonal and independent functionalization of the edges and the basal plane, two methods were attempted (Figure 1). The first method involved edge functionalization with tosylhydrazine (oxo‐GNRs−E1) or iodophenylhydrazine (oxo‐GNRs−E2) followed by surface functionalization with chlorophenyl diazonium salts. The second approach reversed the order, starting with 4‐iodobenzenediazonium salt followed by tosylhydrazine. Figure 2 shows the FTIR spectra of oxo‐GNRs and the corresponding reaction products.

First, we will discuss the edge‐functionalized samples oxo‐GNRs−E1 (functionalized with tosylhydrazine) and oxo‐GNRs−E2 (functionalized with 4‐iodophenylhydrazine). As expected, the intensity of the carbonyl band[^17^, ^18^] at 1730 cm^−1^ in the functionalized samples decreases noticeably compared to that of the pristine oxo‐GNRs. This indicates that the amount of ketone groups is significantly decreased by edge‐functionalization, which was likely due to the reaction of diketones with hydrazine derivatives, while the lactones on the rims identified in our earlier study^[13]^ remain unaffected. Aliphatic C−H bands emerge at 2920 cm^−1^ and 2859 cm^−1^ after the reaction, which may originate from the methyl group of the tosylate and we speculate that C−H bonds are formed due to the overall reducing effect of hydrazines on the oxo‐GNRs.[^19^, ^20^] The C=C band^[18]^ around 1590 cm^−1^ remains largely unaffected. Newly formed C=N[^21^, ^22^] and C−N[^23^, ^24^] bands at 1500 cm^−1^ and 1080 cm^−1^ of the functionalized sample confirm the emergence of a pyrazoline‐like structure at the edges of oxo‐GNRs. Tosylamide was used as reference compound, which exhibits a characteristic and strong S=O stretching vibration, which is observed at about 1160 cm^−1^ for tosylamide and 1163 cm^−1^ for oxo‐GNRs−E1. The weaker vibration at 1360 cm^−1^ in tosylamide,^[25]^ is however not detected in oxo‐GNRs−E1, probably being masked by other vibrations. In the following, we will discuss the basal plane functionalized sample oxo‐GNRs−B, which was functionalized with 4‐iodbenzediazonium salt. The introduction of the iodophenyl group leads to a higher intensity of the C=C band relative to the C=O band in the FTIR spectrum (Figure 2). In addition, C−H vibrations of the phenyl ring appear prominently at 2800 cm^−1^ and 1100 cm^−1^. Compared to oxo‐GNRs, the FTIR spectra of both orthogonal functionalized samples (oxo‐GNRs−O1 and oxo‐GNRs−O2) display all features associated with attached pyrazoline and aryl groups, such as C=N, C−N, and C−H. Additionally, the intensity of the C=O band relative to the C=C band is reduced compared to unfunctionalized oxo‐GNRs, further indicating successful functionalization. The sample functionalized with tosylhydrazine (oxo‐GNRs−O2) also exhibits the characteristic S=O vibration at approximately 1160 cm^−1^. Table 1 presents the elemental compositions of all samples derived from XPS spectra, along with calculations of the proportion of functionalized edge versus basal plane carbon atoms based on a structural model for oxo‐GNRs previously reported.^[13]^ In the edge‐functionalized samples, the proportion of functionalized carbon atoms ranges from 10 % to nearly 40 %. In comparison, the surface‐functionalized samples show a lower proportion of functionalized carbon atoms on the basal plane, ranging from approximately 1 % to 4 %. XPS analysis shows a higher C/O ratio for all functionalized samples compared to the precursor oxo‐GNRs. A higher carbon content can be attributed to the introduction of carbon‐rich functional groups, while a lower content of oxygen is due to the reduction reactions. Furthermore, the edge‐functionalized samples (oxo‐GNRs−E1, oxo‐GNRs−E2) exhibit increased levels of nitrogen and their respective probe atoms, iodine and sulfur. For each probe heteroatom, multiple distinct species were identified in the high‐resolution XPS spectra. The proportion of each atom was determined by considering only the species that correspond to its reasonable binding state within the pyrazoline or aryl moiety. Further details are provided in the supplementary information. For the iodophenyl‐functionalized sample (oxo‐GNRs−E2), the nitrogen‐to‐iodine ratio of approximately 2 is consistent with the structural model shown in Figure 1. In contrast, this ratio in the tosyl‐functionalized sample (oxo‐GNRs−E1) is higher than expected, which might be due to the partial hydrolysis of the formed tosylated pyrazoline,[^26^, ^27^] leading to the cleavage of the tosyl moiety and resulting in unsubstituted pyrazoline residues at the edges of the oxo‐GNRs. The iodophenyl‐diazonium‐functionalized sample oxo‐GNRs−B shows an increased iodine content and, unexpectedly, also shows a significant amount of nitrogen (1.8 at %). Since diazonium salts are stable in aqueous solutions only below 5 °C^[28]^ and the reaction mixtures were handled at room temperature after functionalization, during work‐up, the nitrogen is most likely not originating from unreacted diazonium salts. Possibly, a portion of the diazonium salt reacts with other components in the reaction mixture, generating impurities that become physisorbed and contribute to the nitrogen content of the final product. As oxo‐GNRs are known to form web‐like structures and tightly entangled networks,^[13]^ it is possible that impurities remain trapped and are not fully removed, even after several days of dialysis.

**Table 1:** Elemental composition and ratios from XPS analysis for functionalized oxo‐GNRs (N/Hetero specifically only refers to the heteroatoms attached to the pyrazoline group).

Sample	Functionalization		Composition (at %)						Ratios		Funct. Atoms (%at)	
	Step 1	Step 2	C	O	N	S	I	Cl	C/O	N/Hetero	Edge	Basal Plane
oxo‐GNRs	/	/	74.1	24.9	0.5	0.1	0.0	0.0	3.0	/	/	/
oxo‐GNRs−E1	Tos	/	76.7	19.6	2.6	0.5	0.0	0.0	3.9	5.2 (S)	12.5 %	/
oxo‐GNRs−E2	I‐Phy	/	78.3	15.3	3.1	0.0	1.5	0.0	5.1	2.1 (I)	37.5 %	/
oxo‐GNRs‐B	I–Dia	/	75.9	19.3	1.8	0.0	1.8	0.0	3.9	/	/	3.5 %
oxo‐GNRs−O1	I‐Phy	Cl‐Dia	74.4	19.9	3.1	0.0	1.3	0.3	3.7	2.4 (I)	31 %	1.2 %
oxo‐GNRs−O2	I–Dia	Tos	85.7	10.6	0.7	0.6	0.6	0.0	8.1	1.2 (S)	10.9 %	2.3 %
Tos=Tosylhydrazine					I–Dia=4‐Iodobenzediazonium salt							
I‐Phy=4‐Iodophenylhydrazine					Cl‐Dia=4‐Chlorobenzediazonium salt							

The orthogonally functionalized sample, first treated with 4‐iodophenylhydrazine (oxo‐GNRs−O1) followed by 4‐chlorobenzediazonium salt shows an increased content of carbon, nitrogen, and the probe atoms iodine and chlorine, indicating successful functionalization of both the edges and the basal plane. In the sample which was functionalized first with 4‐iodobenzediazonium salt followed by tosylhydrazine (oxo‐GNRs−O2), the C/O ratio is remarkably high, while the nitrogen content is lower than expected, particularly in relation to the N/hetero ratio which should be approximately 2. These results suggest that for orthogonal functionalization of oxo‐GNRs, it is more effective to functionalize first with hydrazine derivatives and then with diazonium salts. There is a possibility that hydrazine derivatives may react with oxygen‐containing groups other than diketones. For instance, ketones and phenylhydrazines can react to generate substituted hydrazones.^[29]^ Although hydrazone formation cannot be completely ruled out, the conditions used here, including an aqueous solution and elevated temperature, are expected to cause hydrolysis, reverting any hydrazones to their starting components.[^26^, ^30^] Lactone cleavage by hydrazines is also possible but would require harsh reaction conditions, such as prolonged refluxing, which were not applied in this study.^[31]^

## Conclusions

In conclusion, we have demonstrated the orthogonal functionalization of oxo‐GNRs fabricated by oxidative unzipping of single‐wall CNTs followed by reactions with hydrazine derivatives preferably reacting with ketones located at the rims of oxo‐GNRs and diazonium salts, which react with the π‐surface. The FTIR and XPS analyses confirmed the introduction of pyrazoline and aryl moieties using heteroatoms as labels, which allow quantification by determining elemental ratios. This method was found to functionalize up to 40 % of edge atoms and up to 4 % of basal plane atoms, as indicated by detectable heteroatoms as probes. The sequential functionalization approach, first with phenylhydrazine derivatives and then with diazonium salts, proved to be more effective, compared to the reverse approach. This method offers a versatile and scalable strategy for modifying oxo‐GNRs, enhancing their potential for applications in nanoelectronics. Due to the high proportion of edge functionalities in oxo‐GNRs and the relatively small size of the basal plane, it is possible to detect edge functionalities, which is not possible for graphene oxide flakes, for example. However, we suggest that the presented functionalization can also be applied to the edges of graphene oxide^[32]^ or graphene with oxidatively etched pores.[^33^, ^34^, ^35^]

## Supporting Information Summary

The authors have cited additional references within the Supporting Information.[^12^, ^13^, ^36^, ^37^, ^38^, ^39^, ^40^, ^41^, ^42^, ^43^, ^44^, ^45^, ^46^, ^47^, ^48^, ^49^, ^50^, ^51^, ^52^, ^53^, ^54^, ^55^]

## Conflict of Interests

The authors declare no conflict of interest.

1

## Supporting information

As a service to our authors and readers, this journal provides supporting information supplied by the authors. Such materials are peer reviewed and may be re‐organized for online delivery, but are not copy‐edited or typeset. Technical support issues arising from supporting information (other than missing files) should be addressed to the authors.

Supporting Information

## Data Availability

The data that support the findings of this study are available in the supplementary material of this article.
