# Supplementary material for: Orthogonal Functionalization of Oxo‐Graphene Nanoribbons
Source: Chemistry. 2024 Dec 2;31(5):e202403645. doi: 10.1002/chem.202403645 (PMC11753386; doi:10.1002/chem.202403645)
Supplement: Supplementary file 1 — Supporting Information [file CHEM-31-e202403645-s001.pdf]

Chemistry–A European Journal

Supporting Information

Orthogonal Functionalization of Oxo-Graphene Nanoribbons

Lucia Merkel, Christof Neumann, Christian E. Halbig, Anton Habel, Xin Chen, Andrey Turchanin, and Siegfried Eigler*

Orthogonal Functionalization of Oxo-Graphene Nanoribbons

Lucia Merkel [a], Christof Neumann [b], Christian E. Halbig [a], Anton Habel [a], Xin Chen [a], Andrey Turchanin [b] and Siegfried Eigler *[a]

[a] Institute of Chemistry and Biochemistry, Freie Universität Berlin, Altensteinstraße 23a, 14195 Berlin, Germany, E-mail: siegfried.eigler@fu-berlin.de

[b] Institute of Physical Chemistry, Friedrich Schiller University Jena, Lessingstraße 10, 07743 Jena, Germany

WILEY-VCH

Supporting Information: Orthogonal Functionalization of Oxo-Graphene Nanoribbons

S1. Materials and synthetic methods

General information

Purification *via* dialysis was performed employing SpectraPor pre-wetted RC tubing from Repligen with a weight cut-off of 10 kDa. If not stated otherwise, all chemicals were used as received without further purification.

Preparation of oxo-GNRs

A detailed description of the oxidative unzipping of (6,5)-SWCNTs, including experimental details, has been published elsewhere.[1] Briefly, as-received CNTs (Sigma-Aldrich) were suspended in 98% sulfuric acid, and $KMnO_4$ was added under ice cooling. The mixture was stirred at room temperature in a PTFE reactor and then quenched with 5% H_2O_2. The resulting dispersion was washed three times with deionized water using an ultracentrifuge (2 h, RCF = 73,758 g, Hitachi CS150NX), followed by purification for three days by dialysis with 10 kDa cut-off tubing.

Synthesis of 4-iodobenzenediazonium tetrafluoroborate

4-Iodobenzenediazonium tetrafluoroborate was synthesized according to a procedure first described by Cornilleau *et al.*[2] with modified reaction conditions. A solution of fluoroboric acid (3.00 mL, 17.0 mmol, 1.70 equiv.) was added to a solution of 4-iodoaniline (2.19 g, 10.0 mmol, 1.00 equiv.) in water (3.00 mL). The mixture was cooled down to 0 °C. Then an ice cooled solution of sodium nitrite in water (3.00 mL, 10.2 mmol, 1.02 equiv.) was slowly added into the mixture and stirred at 0 °C for 0.5 h. The precipitate was collected by filtration and washed with 2 mL of cold water. The solid was re-dissolved into a minimum amount of acetone. Subsequently, colourless crystals precipitated by gradual addition of diethyl ether. The resulting product was obtained as thin grey needles. Yield: 1.35 g (43%). ^{1}H NMR (600 MHz, DMSO-d_6): δ [ppm] = 8.43 (d, *J* = 8.9 *Hz*, 2H), 8.35 (d, *J* = 8.9 *Hz*, 2H) ^{13}C NMR (151 MHz, DMSO-d_6): δ [ppm] = 140.25, 132.88, 115.20, 113.70. MS (+ESI): m/z = 230.9438 [M^+] (calc. 230.9414).

WILEY-VCH

Synthesis of 4-chlorobenzenediazonium tetrafluoroborate

4-Chlorobenzenediazonium tetrafluoroborate was synthesized according to a procedure first described by Cornilleau *et al.*[2] with modified reaction conditions. A solution of fluoroboric acid (3.00 mL, 17.0 mmol, 1.70 equiv.) was added to a solution of 4-chloroaniline (1.28 g, 10.0 mmol, 1.00 equiv.) in water (3.00 mL). The mixture was cooled down to 0 °C. Then an ice cooled solution of sodium nitrite in water (3.00 mL, 10.2 mmol, 1.02 equiv.) was slowly added into the mixture leading to the formation of a yellow foam and stirred at 0 °C for 2 h. The foamy precipitate was collected by filtration and washed with 2 mL cold water and three times with 2 mL acetone. The solid was re-dissolved in a minimum amount of acetone. Subsequently, colourless crystals precipitated by gradual addition of diethyl ether. The resulting product was obtained as yellow crystals. Yield: 920 mg (40%). MS (+ESI): m/z = 139.0770 [M+] (calc. 139.01).

On-plane functionalization with benzediazonium tetrafluoroborates

In a typical procedure, 0.22 mmol of the diazonium salt was added to 7 mL of a 0.2 mg/mL dispersion of oxo-GNRs in water under ice cooling. Then, 0.5 mL of acetone was added to improve the solubility of the diazonium salt. The mixture was stirred for 1 h under ice cooling and then for another hour at room temperature. The resulting dispersion separated by an ultracentrifuge (2 h, RCF = 73,758 g) and washed three times by redispersion and ultracentrifugation (2 h, RCF = 73,758 g) in a 1:1 water-acetone mixture. Afterwards the precipitate was redispersed again with the help of brief ultrasonication and further purified by three days of dialysis in a 1:1 water-acetone mixture using 10 kDa cut-off tubing. The functionalization in DMSO was performed analogously at 15°C and 25°C, respectively. Subsequent dialysis was conducted in water. The sample oxo-GNRs-BA was then annealed under vacuum at 200°C for 2 hours.

Edge functionalization with phenylhydrazine derivatives

WILEY-VCH

In a typical procedure, 0.5 mmol of the respective phenylhydrazine derivative was added to 7 mL of a 0.2 mg/mL oxo-GNR dispersion, and the mixture was stirred for one day at 80 °C. The resulting dispersion was purified by three cycles of ultracentrifugation and redispersion in water (4 h at 73,758 g), followed by three days of dialysis in purified water.

S2. Instrumentation

Centrifugation was carried out with a Hitachi CP100NX ultracentrifuge equipped with a P55ST2 (r = 8.40 cm) swing-bucket rotor. For XPS sample preparation, the oxo-GNRs dispersion was drop-casted onto Si/Au wafers and allowed to air-dry. To acquire FTIR spectra, the dispersions were drop-casted onto ZnSe windows from Korth Kristalle and then dried overnight in an oven at 150 °C. The FTIR transmission spectra were subsequently recorded using a Perkin Elmer Spectrum Two FT-IR Spectrometer. X-ray photoelectron spectroscopy was performed using a K-Alpha X-ray Photoelectron Spectrometer System (Thermo Fisher Scientific) with a monochromatic X-ray source (Al K_α) with a spot diameter of 400 μm and an electron detector with 0.5 eV energy resolution. The spectra were calibrated using the Au $4f_{7/2}$ peak (84.0 eV) of the substrate and fitted using Voigt functions after background subtraction. XPS measurements for samples oxo-GNRs-BA, oxo-GNRs-B2 and oxo-GNRs-B3 were conducted using an EnviroESCA spectrometer (SPECS Surface Nano Analysis GmbH, Berlin, Germany) equipped with a monochromatic Al Kα X-ray source (excitation energy: 1486.71 eV) and a PHOIBOS 150 electron energy analyzer. Spectra were recorded in normal emission with a source-to-sample angle of 60°. All measurements were performed in fixed analyzer transmission (FAT) mode. The instrument's binding energy scale was calibrated according to the procedure provided by SPECS Surface Nano Analysis GmbH, following ISO 15472. For quantification, survey spectra were acquired at a pass energy of 80 eV, and the quantification was based on empirical sensitivity factors provided by SPECS, corrected using the spectrometer's transmission function. Raman characterizations were conducted using a confocal Raman microscope (Horiba Xplora Plus, 532 nm excitation wavelength, 0.7 μm laser spot, 1200 grating, 0.5 mW, 100x objectives). AFM images were recorded with an JPK NanoWizard 4 equipped with Tap300-G AFM probes in intermitting contact mode.

S3. XPS data and analysis

WILEY - VCH

S3.1 Fitting procedure, metrics and analysis of C(1s) high resolution spectra

To determine the nature and ratio of various carbon-oxygen bonding states in (functionalized) oxo-GNRs, three components (C=C, C-O, and C=O bonds) were fitted as independent peaks from the C 1s spectra as shown in **Figure S1** for oxo-GNRs, while the survey spectrum is shown in **Figure S2**. The C=C peak usually centered at a binding energy of 284.4 eV was fitted with an additional asymmetry factor of approximately 0.14 to account for the semimetallic character of graphene-like materials, caused by interactions of core holes with electrons in the valence band.[3]

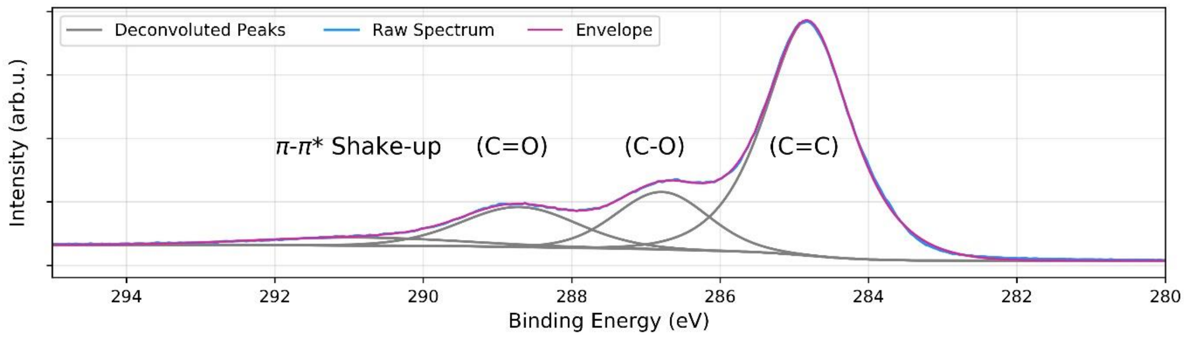

Figure S1: Deconvoluted high-resolution C 1s XPS spectrum of oxo-GNRs with annotated components.

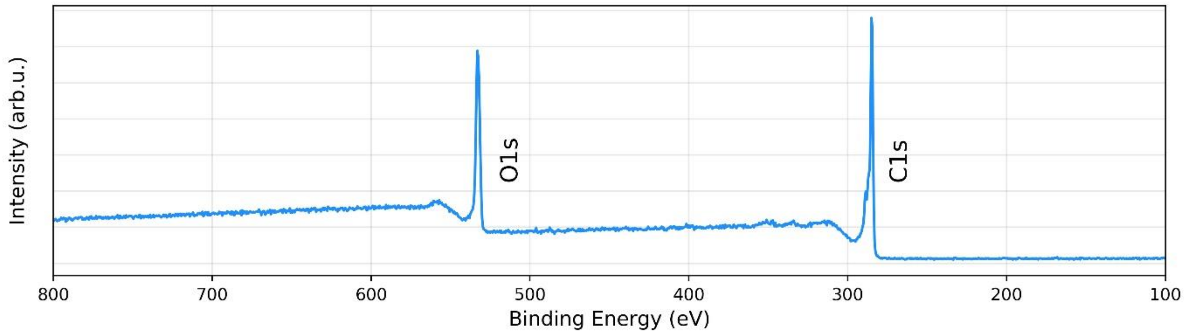

Figure S2: Survey XPS spectrum of oxo-GNRs with annotated peaks.

Changes in the individual component peaks and the resulting C/O ratio provide insights into the success of the functionalization processes. For functionalization with hydrazine derivatives, a decrease in the C=O peak intensity is expected, as hydrazine reacts with 1,3-diketones at the

rims of oxo-GNRs. In contrast, for surface functionalization with diazonium salts, the assessment is less straightforward. Here, the theoretical increase in the C/O ratio due to the introduced aryl group serves as an indicator. **Table S1** summarizes the proportions of different bonding modes and the C/O ratio for the starting material, oxo-GNRs, and the various functionalization products.

Table S1 Deconvoluted components and C/O ratios from C 1s XPS spectra for oxo-GNRs and functionalized reaction products

Sample	C=C		C-O		C=O		C/O Ratio
	Peak (eV)	%	Peak (eV)	%	Peak (eV)	%	
oxo-GNRs	284.8	74.2	286.8	12.0	288.8	11.6	3.0
oxo-GNRs-E1	284.8	82.8	286.4	11.6	288.9	5.6	3.9
oxo-GNRs-E2	284.7	83.9	286.3	11.9	288.8	4.2	5.1
oxo-GNRs-B	284.7	83.5	286.6	9.9	288.5	6.6	3.9
oxo-GNRs-O1	284.7	78.4	286.4	15.8	288.7	5.8	3.7
oxo-GNRs-O2	284.7	92.7	286.3	6.4	288.8	0.9	8.1

S3.2 Comprehensive analysis of species and portions of XPS probe heteroatoms

For some probe heteroatoms, the high-resolution XPS spectra revealed multiple distinct species. The proportion of each atom was calculated by considering only the species corresponding to its plausible binding state within the newly formed pyrazoline or aryl-moiety, respectively. This is discussed in detail below.

oxo-GNRs-E1

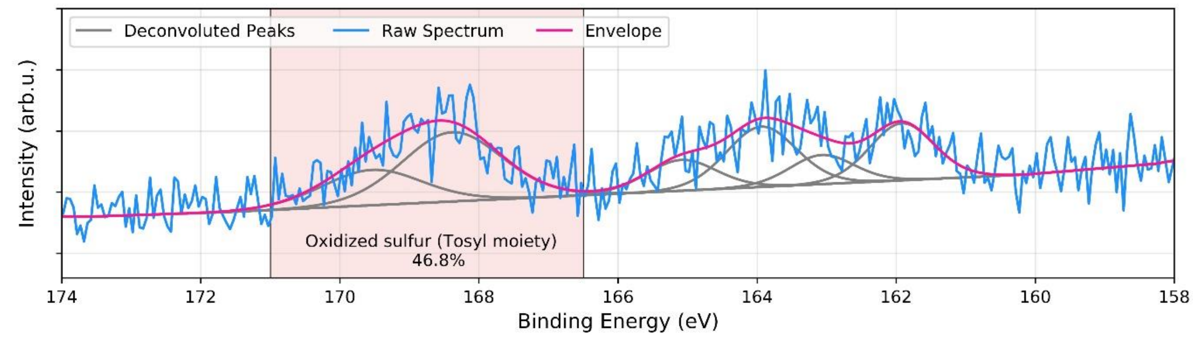

WILEY-VCH

Figure S3: High-resolution S 2p XPS spectrum of oxo-GNRs-E1. The highlighted area refers to the peak belonging to oxidized sulfur and thus, the tosyl-moiety.

The high-resolution S 2p XPS spectrum of oxo-GNRs-E1 shown in **Figure S3** reveals the presence of multiple sulfur species. For determining the sulfur fraction actually bound as tosyl-moiety to the pyrazoline motif, only the peak associated with oxidized sulfur in sulfonates,[4] centered at a binding energy of 168.4 eV and comprising 46.8% of the total sulfur signal, is taken into account. The elemental composition comprises a total sulfur content of 1.0 at%. Considering the proportion of oxidized sulfur, the sulfur specifically bound as tosyl-moiety at the rims of oxo-GNRs is estimated to be 0.5 at%. S $2p_{3/2}$ peaks observed at a binding energy of 163.9 eV and 161.9 eV are likely attributed to thiols[5] and sulfur bound to the gold substrate,[4, 6] respectively. The survey spectrum is shown in **Figure S4**.

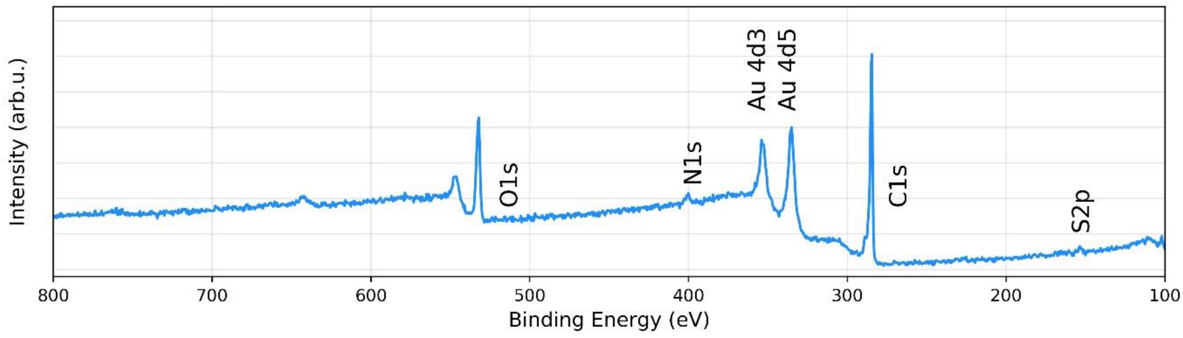

Figure S4: Survey XPS spectrum of oxo-GNRs-E1 with annotated peaks.

oxo-GNRs-E2

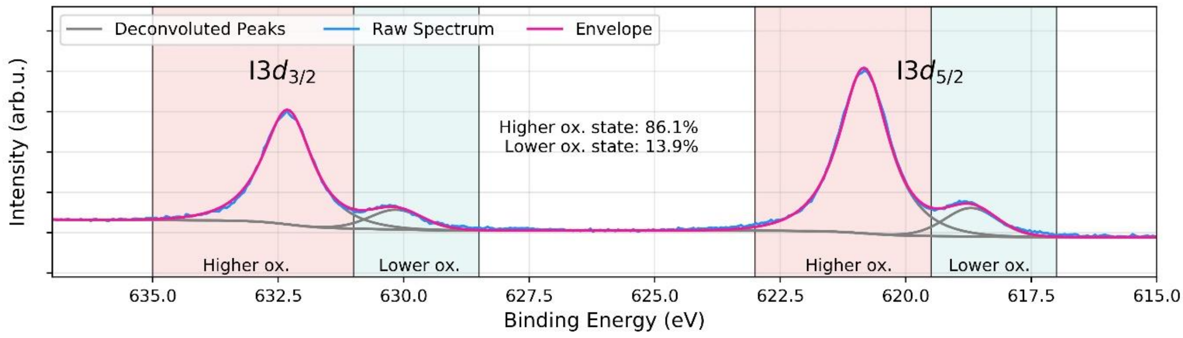

Figure S5: High-resolution I 3d XPS spectrum of oxo-GNRs-E2. Areas highlighted in red and green refer to higher and lower oxidized iodine species found in the sample.

The high-resolution I 3d XPS spectrum of oxo-GNRs-E2 shown in **Figure S5** reveals a characteristic doublet due to spin-orbit splitting within iodine's *d* orbital, showing a peak separation of approximately 11 eV.[7] The peaks are centered around 620 eV ($I3d_{5/2}$) and 632 eV ($I3d_{3/2}$). Each of these peaks is further split into two distinct peaks, indicating the presence of two chemically different iodine species within the sample. The higher oxidized iodine species, centered at a binding energy of 620.8 eV and 632.3 eV, constitutes 86.1% of the total iodine. This species is identified as organically covalently bound iodine, attached to the phenyl group and hence, the newly formed pyrazoline group. The lower oxidized iodine species, with peaks at a binding energy of 618.7 eV and 630.0 eV, represents 13.9% of the total iodine and is suspected to be an impurity, potentially from an ionic iodine salt such as CaI_2 or NaI.[8] To determine the iodine portion bound to the edges of oxo-GNRs and thus, the correct elemental composition of the sample, only the fraction of the higher oxidized species is taken into account. The elemental composition indicates an iodine content of 2.1 at%. Given that 86.1% of the iodine is identified as the relevant chemically bound species, the actual fraction of iodine incorporated into the pyrazoline groups of the oxo-GNRs is calculated to be 1.8 at%.

The high-resolution N 1s spectrum is shown in **Figure S6**. This analysis, exemplified for sample oxo-GNRs-E2, applies to all samples functionalized with a hydrazine derivative, as they all exhibit the same fundamental structural model of a substituted pyrazoline group. No deviations were observed in the N 1s spectra of the other hydrazine-functionalized samples.

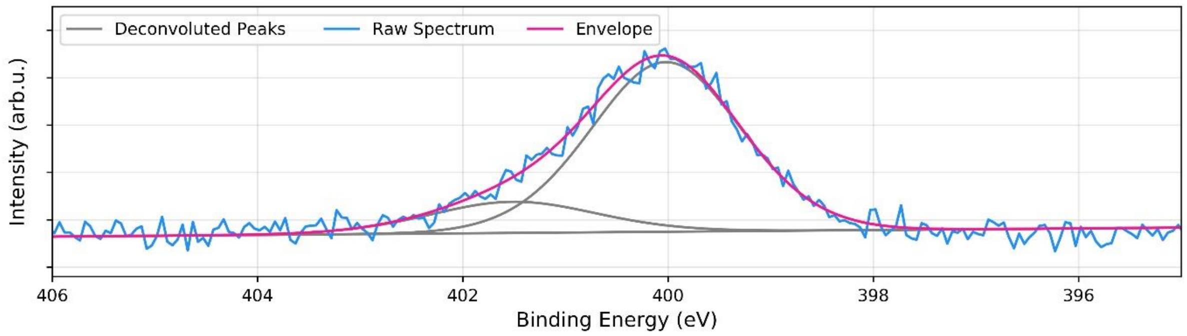

Figure S6: High-resolution N 1s XPS spectrum of oxo-GNRs-E2.

The N 1s spectrum of oxo-GNRs-E2 exhibits two distinct peaks. The larger peak, centered at a binding energy of 400.2 eV, is attributed to both nitrogen atoms of the pyrazoline motif. Despite

the non-identical nature of these nitrogen atoms, their N 1s peaks appear at nearly the same position, resulting in an overlapping peak.[9] The smaller peak at 401.8 eV is likely due to protonated nitrogen atoms.[10] The survey spectrum is shown in **Figure S7**.

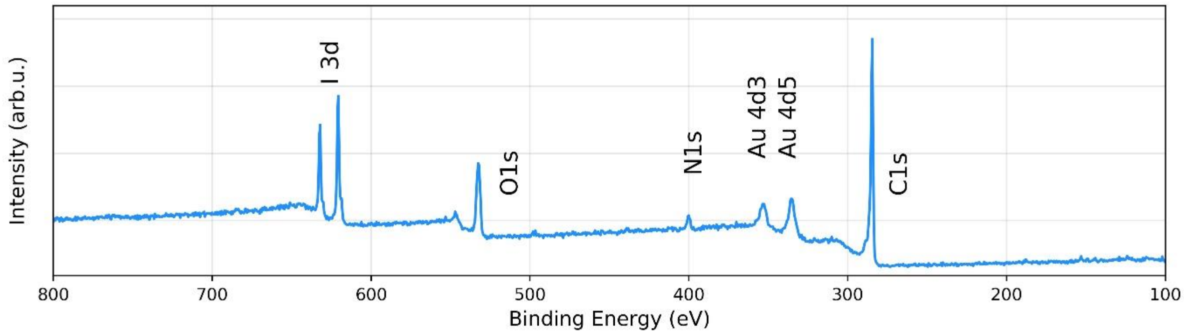

Figure S7: Survey XPS spectrum of oxo-GNRs-E2 with annotated peaks.

oxo-GNRs-B

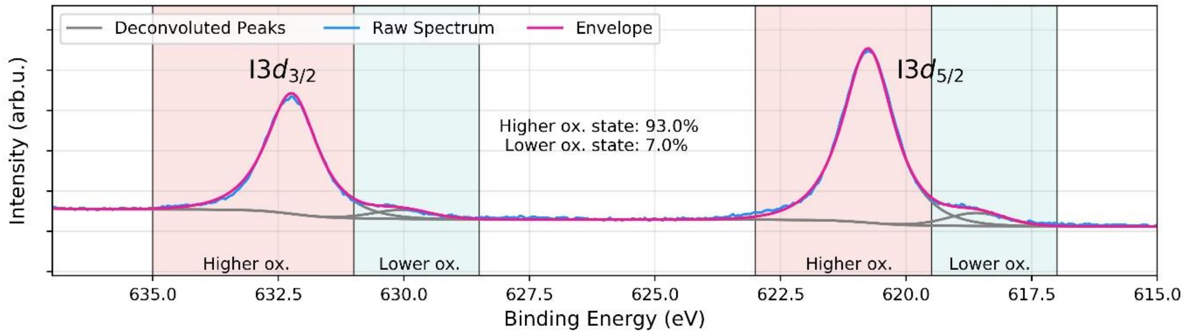

Figure S8: High-resolution I 3d XPS spectrum of oxo-GNRs-B. Areas highlighted in red and green refer to higher and lower oxidized iodine species found in the sample.

In the sample functionalized on-plane using iodine diazonium salt, two distinct iodine species were identified as shown in **Figure S8**. The iodine fraction taken into account for the elemental composition was calculated analogously to the oxo-GNRs-E2 sample. The survey spectrum is shown in **Figure S9**.

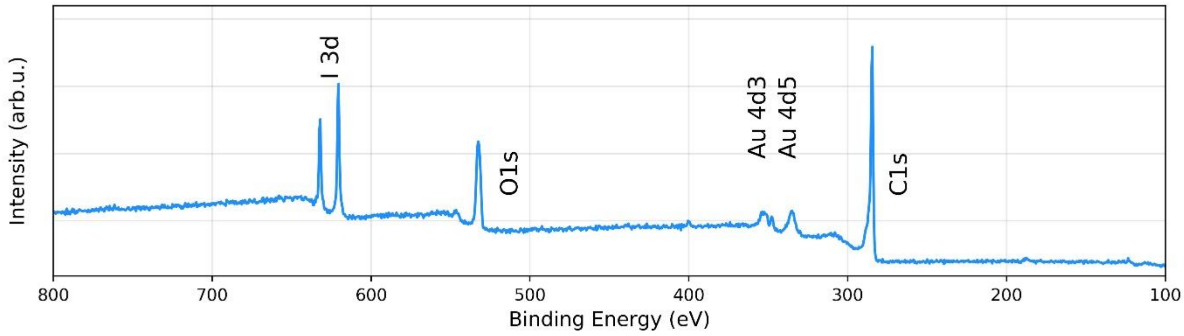

Figure S9: Survey XPS spectrum of oxo-GNRs-B with annotated peaks.

oxo-GNRs-O1

Sample oxo-GNRs-O1 was orthogonally functionalized, with the initial step involving the functionalization of the edges using a 4-iodophenylhydrazine. Here, two distinct iodine species were found, evident in **Figure S10**. The iodine fraction taken into account for the elemental composition was calculated analogously to the oxo-GNRs-E2 sample.

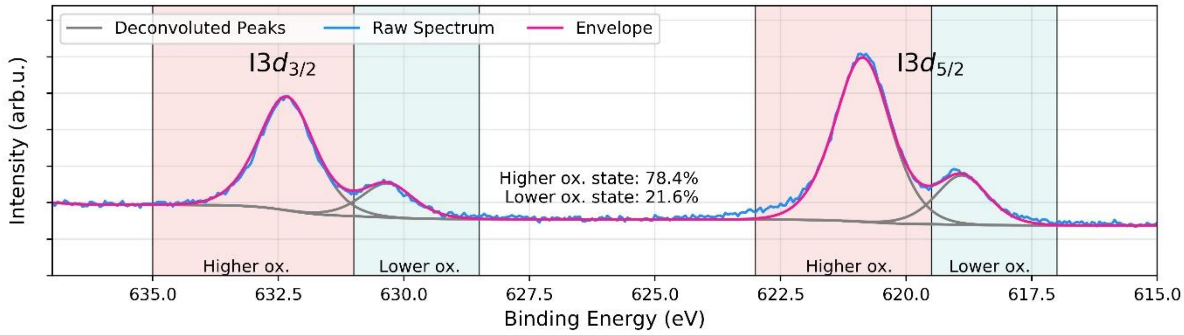

Figure S10: High-resolution I 3d XPS spectrum of oxo-GNRs-O1. Areas highlighted in red and green refer to higher and lower oxidized iodine species found in the sample.

In the second step, oxo-GNRs-O1 were functionalized on-plane using a chlorodiazonium salt. **Figure S11** presents the high-resolution Cl 2p spectrum of the sample, while the survey spectrum is shown in **Figure S12**. While the intensity of the chlorine peak is to low to be seen in the survey spectrum, it appears in the high resolution spectrum. Only one chlorine species is visible at a binding energy of 200.7 eV, indicating organic chlorine bound to the aryl group. The observed peak is split into two components due to spin-orbit splitting within chlorine's *p* (Cl $2p_{3/2}$ and Cl $2p_{1/2}$) orbitals with a typical peak separation of approximately 1.6 eV.[11] The

peak centered around 188 eV stems from the 4s orbital of iodine,[12] which was introduced in the first synthesis step.

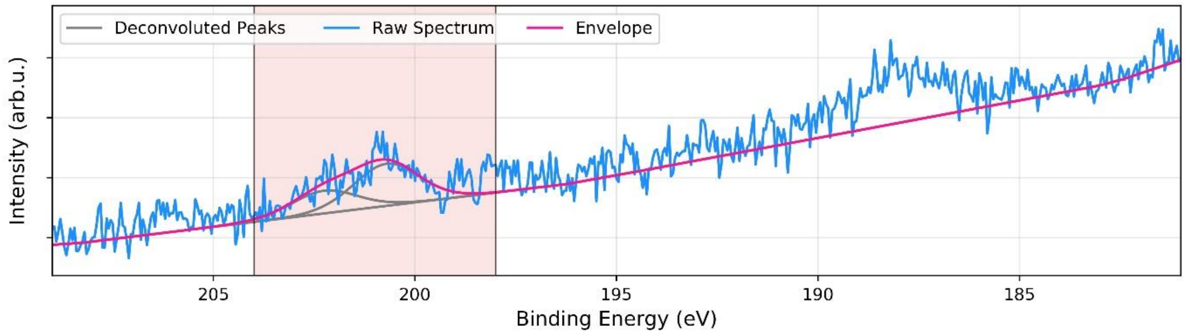

Figure S11: High-resolution Cl 2p XPS spectrum of oxo-GNRs-O1. The area highlighted in red refers to the relevant chlorine peak.

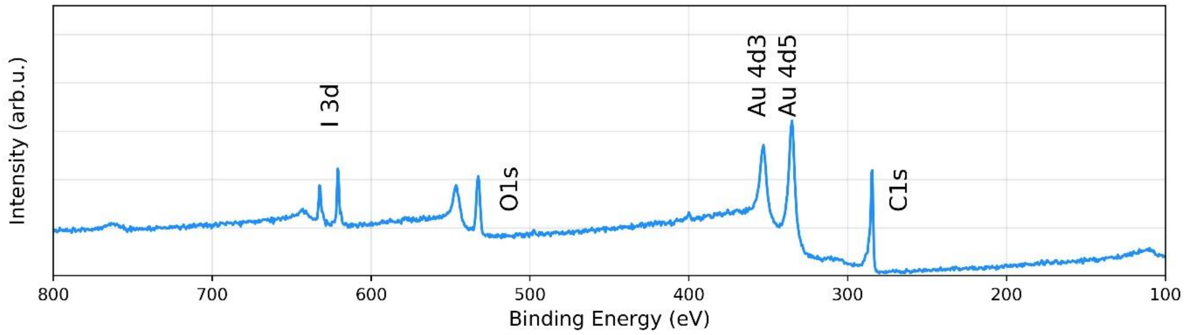

Figure S12: Survey XPS spectrum of oxo-GNRs-O1 with annotated peaks.

oxo-GNRs-O2

Sample oxo-GNRs-O2 was orthogonally functionalized, with the initial step involving the functionalization of the surface using a iododiazonium salt. **Figure S13** presents the high-resolution I 3d XPS spectrum of the sample. The iodine fraction taken into account for the elemental composition was calculated analogously to the oxo-GNRs-E2 sample.

WILEY

WILEY-VCH

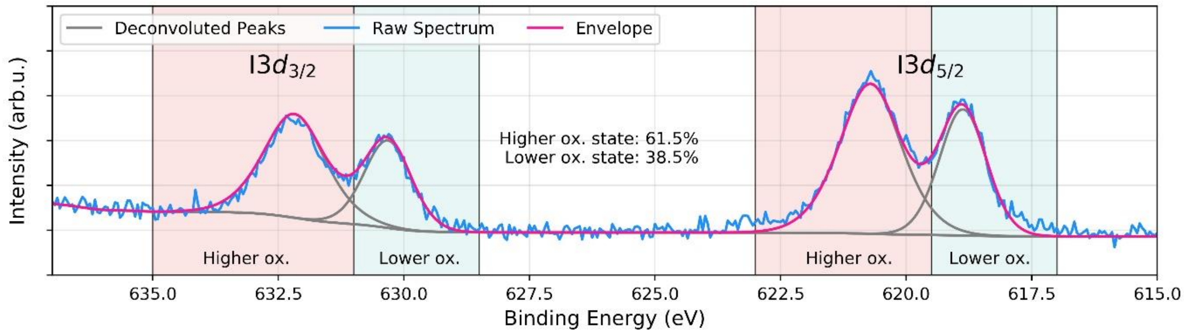

Figure S13: High-resolution I 3d XPS spectrum of oxo-GNRs-O2. Areas highlighted in red and green refer to higher and lower oxidized iodine species found in the sample.

The second step involved the edge functionalization with tosylhydrazine. The high-resolution S 2p XPS spectrum of oxo-GNRs-O2 shown in Figure S14 reveals the multiple sulfur species. The sulfur fraction taken into account for the elemental composition was calculated analogously to the oxo-GNRs-E1 sample. The survey spectrum is shown in Figure S15. Here, the intensity of the sulfur peak is too low to be seen in the survey spectrum. However, it appears in the high resolution spectrum.

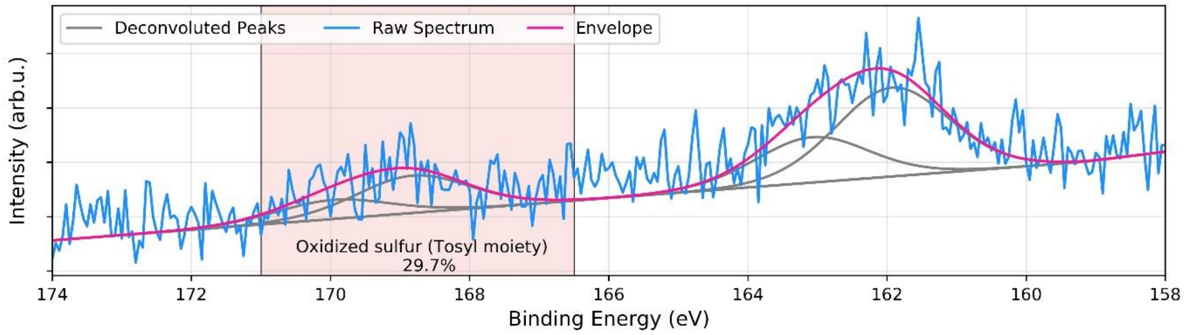

Figure S14: High-resolution S 2p XPS spectrum of oxo-GNRs-O2. The highlighted area refers to the peak belonging to oxidized sulfur and thus, the tosyl-moiety.

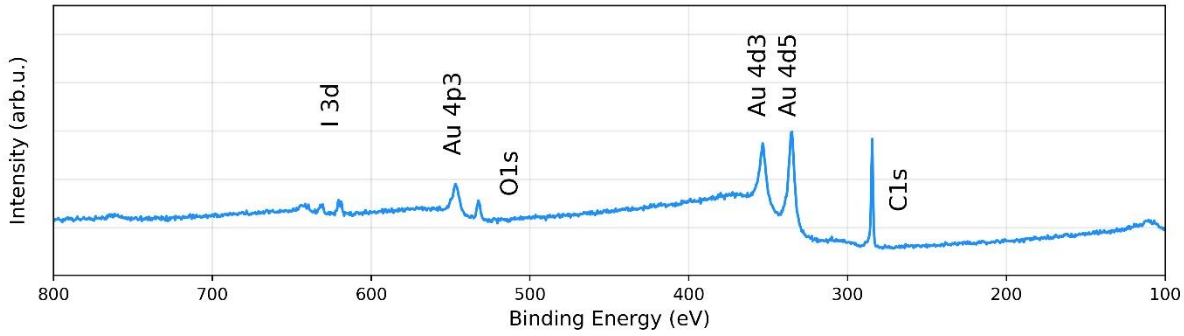

Figure S15: Survey XPS spectrum of oxo-GNRs-O2 with annotated peaks.

S4. Raman analysis

Figure S16 displays the D and G peak areas in the Raman spectra of oxo-GNRs and its functionalized products, with the corresponding metrics from Voigt profile fitting presented in **Table S2**. While the *I*D/*I*G ratio is typically a useful metric for assessing defect density, its relevance is limited for structures as small as these oxo-GNRs. With widths of only 2–4 nm, as demonstrated in previous studies,[1] the oxo-GNRs exhibit an already high defect density due to their small size and edge effects, which contribute substantially to the D peak. Although functionalization introduces additional defects, the already high amount of defect sites cause overlapping scattering effects, leading to minimal changes in the *I*D/*I*G ratio upon functionalization.[13] Another possible metric for assessing defect density is the absolute intensity of the D and G peaks. However, for oxo-GNRs, this is challenging due to their web-like, networked structures with inconsistent thickness and layering.[1] While increased defects typically enhance photoluminescence intensity by creating more individual sp^2 islands, this metric is reliable only with consistent sample thickness, making direct intensity comparisons unreliable for these GNRs.[13] Another metric for assessing defect density is the full width at half maximum (FWHM) of the D and G peaks. Higher FWHM values indicate greater lattice disorder, and thus, increased defect density.[14-15] As shown in **Table S2**, the FWHM of both peaks increases with the degree of functionalization, particularly in the D peak when comparing mono- and double-functionalization, indicating effective functionalization and associated defect introduction.

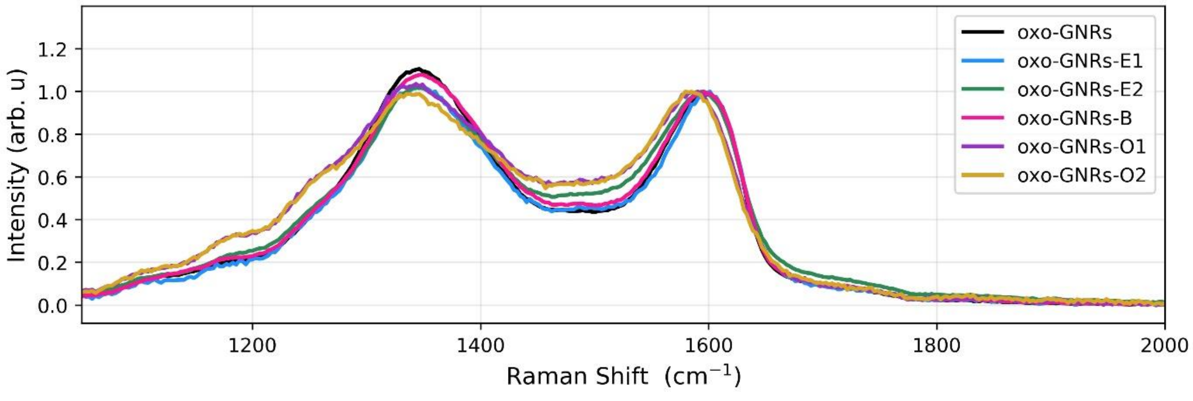

Figure S16: D and G peak area of the Raman spectra of oxo-GNRs and functionalized reaction products.

Table S2 Raman metrics for oxo-GNRs and functionalized reaction products

Sample	*I*D/*I*G	*I* D (cts.)	*I* G (cts.)	FWHM D (cm-1)	FWHM G(cm-1)
oxo-GNRs	1.11	230390	207512	69 ± 12	84 ± 6
oxo-GNRs-E1	1.03	49804	48480	153 ± 10	94 ± 3
oxo-GNRs-E2	1.02	414038	405911	190 ± 9	100 ± 6
oxo-GNRs-B	1.08	356451	330475	129 ± 9	95 ± 4
oxo-GNRs-O1	1.17	60220	51319	216 ± 10	99 ± 2
oxo-GNRs-O2	1.00	40952	41010	224 ± 8	94 ± 11

S5. AFM analysis of oxo-GNRs-B

Covalent functionalization of graphene increases its thickness, which can be effectively measured using atomic force microscopy. Diazonium salts, commonly used for graphene functionalization, are highly reactive and often lead to polymeric multilayer formation or even dendritic growth due to their radical nature. These radicals bond to the graphene surface but can also react with previously grafted layers, further increasing thickness.[16-17] To confirm successful functionalization, we investigated whether the thickness of oxo-GNRs changes after treatment with iodophenyldiazonium salts (oxo-GNRs-B). An increase in thickness would indicate effective grafting of aryl groups onto the GNR surface. **Figure S17** shows AFM images and height profiles of the unfunctionalized oxo-GNRs used as the starting material. The ribbons exhibit a height of approximately 1 nm, which is consistent with the expected values for oxo-graphene. **Figure S18** presents AFM images and height profiles of the diazonium-functionalized sample, oxo-GNRs-B, where a noticeable increase in thickness of roughly 4 nm to 6 nm is observed across all measured areas. Greenwood *et al.* highlighted that the choice of diazonium salt significantly affects the formation of polymeric grafts or multilayers.[17] They reported a monolayer thickness of 0.67 nm with 3,5-bis-tert-butylbenzenediazonium salt, whereas 4-nitrobenzenediazonium salt produced multilayered structures with a thickness of 3.6 nm. The evidence strongly suggests that the 4-iodophenyldiazonium salt used in this study also tends to form polymeric layers, which could explain the increased thickness of the structures observed after functionalization. However, it is important to note that measuring the height of oxo-GNRs using AFM not trivial due to their tendency to form entangled, web-like structures, as reported before,[1, 18] making it challenging to isolate a single nanoribbon for precise measurement.

WILEY-VCH

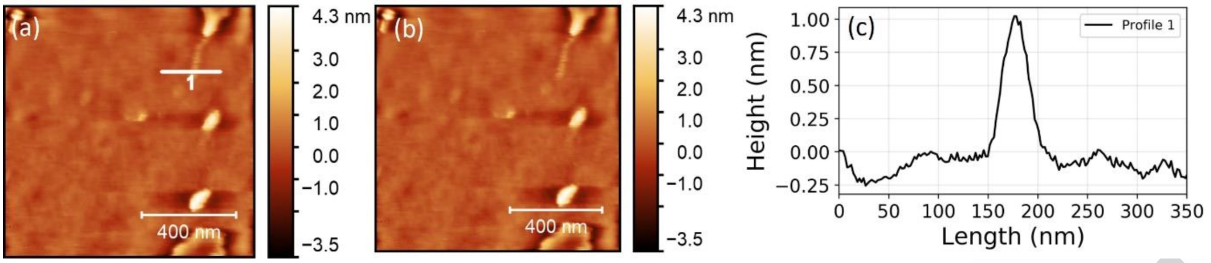

Figure S17: (**a**) AFM image of oxo-GNRs with the height profile line indicated. (**b**) The same AFM image without the height profile line for improved visibility. (**c**) Height profile corresponding to the indicated line in (**a**).

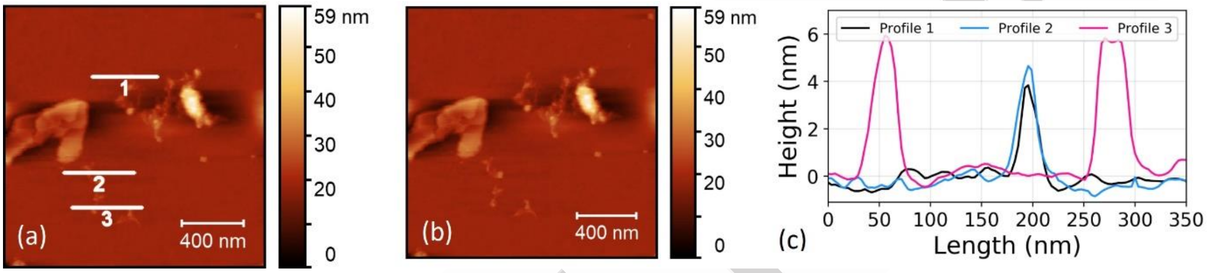

Figure S18: (**a**) AFM image of oxo-GNRs-B with the height profile line indicated. (**b**) The same AFM image without the height profile line for improved visibility. (**c**) Height profile corresponding to the indicated line in (**a**).

S6. Further insights into the diazonium functionalization of oxo-GNRs

Diazonium functionalization of graphene is reversible, for example by thermal desorption achieved through annealing of the material.[19-20] In this study, successful thermal desorption would be indicated by the absence of probe heteroatoms, specifically iodine, initially introduced via diazonium salts. **Table S3** shows the elemental composition of the pristine diazonium-functionalized sample oxo-GNRs-B along with the annealed sample oxo-GNRs-BA.

Table S3 Elemental composition and ratios from XPS analysis for functionalized oxo-GNRs

Sample	Composition (at%)		
	C	O	I
oxo-GNRs-B	75.9	19.3	1.8

WILEY-VCH

oxo-GNRs-BA (Annealed, 2h, 200°C)	75.8	22.4	1.8
oxo-GNRs-B2 in DMSO (25 °C)	81.7	17.0	1.3
oxo-GNRs-B3 in DMSO (15 °C)	78.2	20.8	1.0

The iodine content, initially approximately 1.8, remains unchanged after annealing. Several factors could account for this observation. First, annealing at 200°C for 2 hours may not have been sufficient to induce significant structural changes. Alternatively, the web-like, entangled structures of the oxo-GNRs could explain this observation. Thermal annealing generates aryl radicals, which may become trapped at alternative positions within the material or recombine to form dimers that can physisorb within the entangled structure. Such recombination following the cleavage of covalent bonds on graphene has previously been observed, specifically in the dimerization of hexyl radicals to form dodecane.[20]

The choice of solvent significantly impacts the effectiveness of graphene functionalization with diazonium salts. DMSO, reported as a suitable solvent achieving high functionalization degrees, was also tested in this study to examine potential solvent effects.[21] Depending on reaction temperature, iodine content in DMSO-functionalized samples ranged from 1.0% to 1.3% (oxo-GNRs-B2), slightly lower than the 1.8% (oxo-GNRs-B3) observed with water (oxo-GNRs-B) as shown in **Table S3**. While this difference is insufficient to determine the superior solvent, it confirms that functionalization proceeds effectively in DMSO. The XPS survey spectra are shown in **Figure S19**.

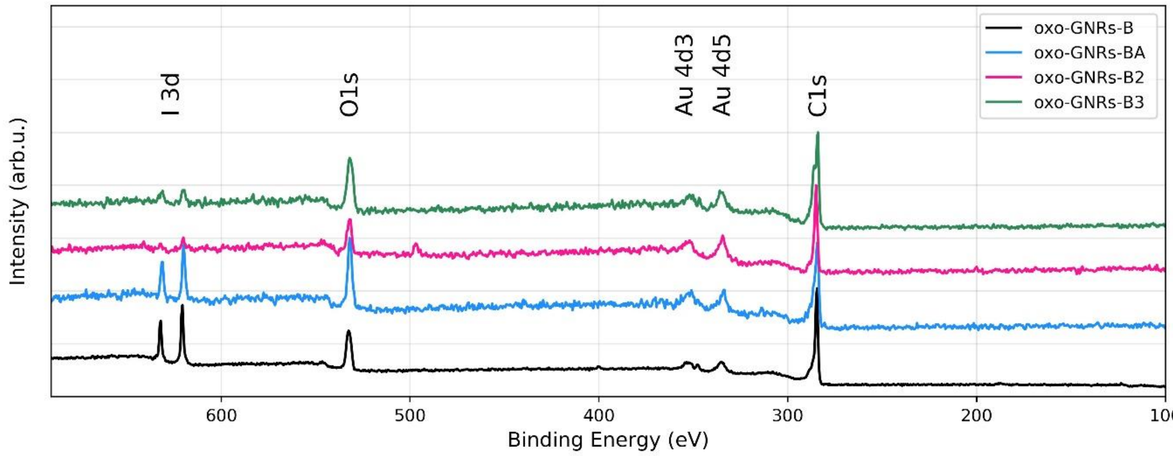

Figure S19: Survey XPS spectrum of diazonium functionalized oxo-GNRs with annotated peaks.

References

[1] L. Merkel, A. Setaro, C. E. Halbig, S. Shimizu, T. Yoshii, H. Nishihara, T. Hilal, G. Algara-Siller, C. Koch, S. Eigler, *Carbon* **2024**, *229*, 119454.
[2] T. Cornilleau, P. Hermange, E. Fouquet, *Chem. Commun.* **2016**, *52*, 10040-10043.
[3] A. Kovtun, D. Jones, S. Dell'Elce, E. Treossi, A. Liscio, V. Palermo, *Carbon* **2019**, *143*, 268-275.
[4] P. Gobbo, M. C. Biesinger, M. S. Workentin, *Chem. Commun.* **2013**, *49*, 2831-2833.
[5] D. G. Castner, K. Hinds, D. W. Grainger, *Langmuir* **1996**, *12*, 5083-5086.
[6] P. Gobbo, Z. Mossman, A. Nazemi, A. Niaux, M. C. Biesinger, E. R. Gillies, M. S. Workentin, *J. Mater. Chem. B* **2014**, *2*, 1764-1769.
[7] R. Forbes, A. De Fanis, C. Bomme, D. Rolles, S. T. Pratt, I. Powis, N. A. Besley, M. Simon, S. Nandi, A. R. Milosavljević, C. Nicolas, J. D. Bozek, J. G. Underwood, D. M. P. Holland, *J. Chem. Phys.* **2018**, *149*, 144302.
[8] H. F. Wang, X. Y. Deng, J. Wang, X. F. Gao, G. Xing, Z. Shi, Z. N. Gu, Y. F. Liu, Y. L. Zhao, *Acta Phys. - Chim. Sin.* **2004**, *20*, 673-675.
[9] S. Park, Y. Hu, J. O. Hwang, E.-S. Lee, L. B. Casabianca, W. Cai, J. R. Potts, H.-W. Ha, S. Chen, J. Oh, S. O. Kim, Y.-H. Kim, Y. Ishii, R. S. Ruoff, *Nat. Commun.* **2012**, *3*, 638.
[10] J. S. Stevens, S. Coultas, C. Jaye, D. A. Fischer, S. L. M. Schroeder, *Phys. Chem. Chem. Phys.* **2020**, *22*, 4916-4923.
[11] I. Bello, W. H. Chang, W. M. Lau, *J. Appl. Phys.* **1994**, *75*, 3092-3097.
[12] B. V. Crist, *Handbook of monochromatic XPS spectra: Handbook of monochromatic XPS spectra elements and native oxides*, John Wiley & Sons, Chichester, England, **2000**.
[13] P. Vecera, S. Eigler, M. Koleśnik-Gray, V. Krstić, A. Vierck, J. Maultzsch, R. A. Schäfer, F. Hauke, A. Hirsch, *Sci. Rep.* **2017**, *7*, 45165.
[14] A. Eckmann, A. Felten, A. Mishchenko, L. Britnell, R. Krupke, K. S. Novoselov, C. Casiraghi, *Nano Lett.* **2012**, *12*, 3925-3930.
[15] L. G. Cançado, A. Jorio, E. H. M. Ferreira, F. Stavale, C. A. Achete, R. B. Capaz, M. V. O. Moutinho, A. Lombardo, T. S. Kulmala, A. C. Ferrari, *Nano Lett.* **2011**, *11*, 3190-3196.
[16] T. M. T. Huynh, K. Tahara, S. De Feyter, T. H. Phan, *RSC Adv.* **2023**, *13*, 24576-24582.
[17] J. Greenwood, T. H. Phan, Y. Fujita, Z. Li, O. Ivasenko, W. Vanderlinden, H. Van Gorp, W. Frederickx, G. Lu, K. Tahara, Y. Tobe, H. Uji-i, S. F. L. Mertens, S. De Feyter, *ACS Nano* **2015**, *9*, 5520-5535.
[18] Z.-S. Wu, W. Ren, L. Gao, B. Liu, J. Zhao, H.-M. Cheng, *Nano Res.* **2010**, *3*, 16-22.
[19] H. Van Gorp, P. Walke, J. Teyssandier, B. E. Hirsch, H. Uji-i, K. Tahara, Y. Tobe, M. Van der Auweraer, S. De Feyter, *J. Phys. Chem. C* **2020**, *124*, 1980-1990.-
[20] C. E. Halbig, O. Martin, F. Hauke, S. Eigler, A. Hirsch, *Chem. Eur. J.* **2018**, *24*, 13348-13354.
[21] Y. Xia, L. Sun, S. Eyley, B. Daelemans, W. Thielemans, J. Seibel, S. De Feyter, *Adv. Sci.* **2022**, *9*, 2105017.

WILEY-VCH
